# Local tumour residue after microwave ablation for lung cancer: a case report

**DOI:** 10.1093/icvts/ivac277

**Published:** 2022-11-21

**Authors:** Guixian Liu, Miqi Gu, Xin Wang, Jintao He

**Affiliations:** Department of Clinical Medicine, Southwest Medical University, Luzhou, China; Department of Clinical Medicine, Southwest Medical University, Luzhou, China; Department of Thoracic Surgery, Sichuan Cancer Hospital, Chengdu, China; Department of Clinical Medicine, Southwest Medical University, Luzhou, China; Department of Thoracic Surgery, Sichuan Cancer Hospital, Chengdu, China

**Keywords:** Pulmonary nodule, Microwave ablation, Residual tumour

## Abstract

Thermal ablation has become a novel method for the treatment of pulmonary nodules, but the short-time evaluation of the ablation effect is mainly based on computed tomography images. We report a case of local tumour residue after microwave ablation, which was confirmed by pathology after lobectomy. This case alerts us that thermal ablation should not be the preferred treatment for operable pulmonary nodules.

## INTRODUCTION

With the wide application of thoracic computed tomography (CT), increasing cases of pulmonary nodules are identified [1]. Thermal ablation (TA) has become a novel method for the treatment of multiple pulmonary nodules. The evaluation of the ablation effect is mainly based on CT images and long-time follow-up. And in previous studies [2], some cases of tumour recurrence after ablation were found during follow-up. We report a case of local tumour residue after microwave ablation, which was confirmed by pathology after lobectomy.

## CASE REPORT

**Figure 1:**
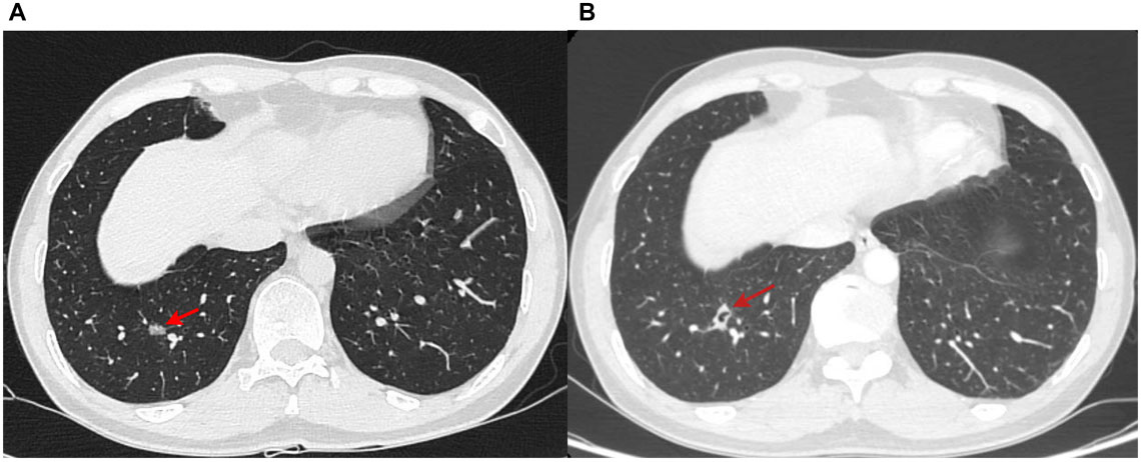
(**A**) Computed tomography image before ablation. (**B**) Computed tomography image of 2 months after ablation.

**Figure 2:**
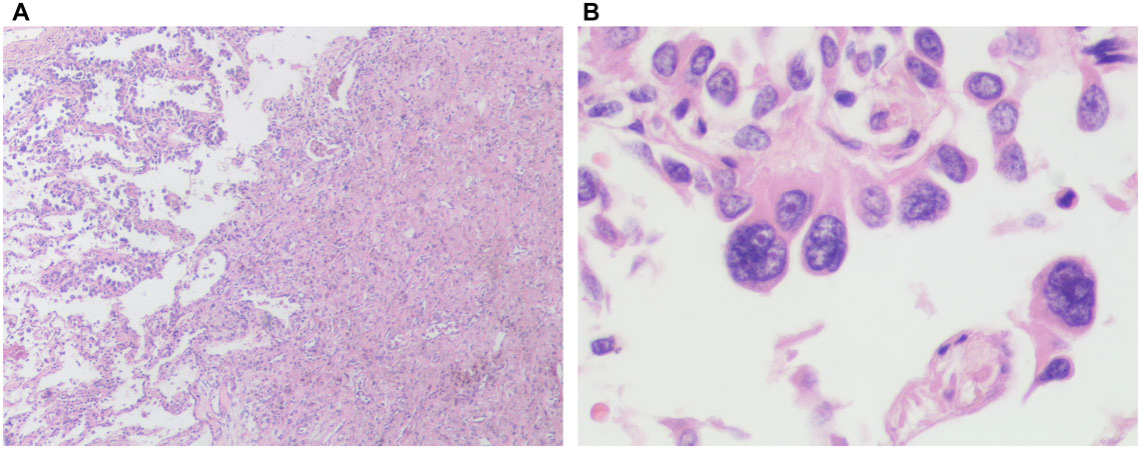
(**A**) The fibrotic area and the heterotypical glands (Hematoxylin-Eosin staining 100×). (**B**) The heterotypical glands (Hematoxylin-Eosin staining 400×).

A 56-year-old male patient was admitted to the local hospital with nodules of the right lower lobe (RLL). CT scan showed a 9-mm slightly high-density nodule with lobulation signs in the posterior basal segment of the RLL (Fig. 1A). Because of the fear of surgery, the patient received CT-guided microwave ablation synchronous biopsy of the nodule on 10 May 2022. The pathological diagnosis was adenocarcinoma. A month later, the CT scan showed a 16-mm nodule in the posterior basal segment of the RLL, and an 8-mm vacuole can be seen in the centre of the nodule. The patient was admitted to our hospital because of extreme anxiety and required surgical removal of nodules. CT scan showed 1.4 cm × 0.7 cm irregular solid nodules in the posterior basal segment of the RLL, and a vacuole can be seen in the nodule (Fig. 1B). After a discussion in the department of thoracic surgery, we removed the RLL on 11 June 2022. Pathological diagnosis revealed that most of the areas were fibrotic, and a few heterotypical glands were found at the edge (about 0.3 cm in diameter), which tended to be adenocarcinoma (Fig. 2).

## DISCUSSION

TA has been used in pulmonary nodules as a minimally invasive treatment. It is possible to achieve curative ablation of subsolid nodules with a maximum diameter of ≤30 mm and a consolidation tumour ratio of ≤50% due to rare lymphatic or distant metastasis [1]. But there is still no high level of evidence that TA can be an alternative to surgery or stereotactic body radiotherapy (SBRT). And compared with surgery, its advantages are mainly short-term benefits due to the minimally invasive method, especially in the elderly and patients with complications [3]. But compared to SBRT, the only non-invasive alternative to surgery, TA still has a higher risk of short-term complications than SBRT, such as gas embolism, haemorrhage, lung abscess, pneumothorax, and haemothorax [3]. In our case, the patient, an able-bodied middle-aged man, had no contraindications to surgery or SBRT, but he refused surgery and SBRT due to fear. In the expert consensus of China, a multidisciplinary team is recommended for discussion and decision-making on the indications of TA, and shared decision-making (SDM) should be carried out if necessary [1]. The effect of ablation is mostly evaluated by imaging examination, there is a high risk of incomplete ablation. To identify the normal imaging signs after ablation, we may have more demand for enhanced CT and positron emission tomography / computed tomography (PET/CT) during the follow-up. One of the advantages of TA is that it can be performed after the biopsy via a coaxial cannula, which allows for simultaneous diagnosis and treatment within a single procedure [4], but if the amount of biopsy tissue is insufficient, we will lose the opportunity to identify the tissue type and complementary biomolecular analyses. For tumour patients, we should comprehensively consider the benefits of patients and should not sacrifice long-term benefits because of minimally invasive or non-invasive.

## Data Availability

No new data were generated or analysed in support of this research. And the figures underlying this article are available in the article and in its online supplementary material.
