# Correction to: Local tumour residue after microwave ablation for lung cancer: a case report

**DOI:** 10.1093/icvts/ivad078

**Published:** 2023-05-24

This is a correction to: Guixian Liu, Miqi Gu, Xin Wang, Jintao He, Local tumour residue after microwave ablation for lung cancer: a case report, *Interactive CardioVascular and Thoracic Surgery*, Volume 35, Issue 6, December 2022, ivac277, https://doi.org/10.1093/icvts/ivac277

In the originally published version of this manuscript, the authors’ affiliations were incomplete. Guixian Liu's affiliation is “Department of Thoracic Surgery, School of Clinical Medicine, Southwest Medical University, China”, and Jintao He's affiliation is “Department of Thoracic Surgery, School of Clinical Medicine, Southwest Medical University, China; Department of Thoracic Surgery, Sichuan Cancer Hospital, China”.

This error has been corrected online.

